# Intra-Rater Reliability of Pressure Pain Threshold with Different Algometers in Healthy Participants

**DOI:** 10.3390/muscles4010005

**Published:** 2025-02-11

**Authors:** Alexandre Nunes, Vanessa Leite

**Affiliations:** 1 Osteopatia, Escola Superior de Saúde Jean Piaget do Algarve, Enxerim, 8300-025 Silves, Portugal; vanessaleittee@gmail.com; 2 Insight: Centro de Investigação Piaget para o Desenvolvimento Humano e Ecológico, 1950-157 Lisboa, Portugal; 3 Portugal National Centre, Foundation COME Collaboration, 8125-196 Quarteira, Portugal

**Keywords:** pressure pain threshold, digital algometer, intra-reliability

## Abstract

Background: Algometry is a validated and reliable measurement tool, but there are still no reliable data for the different algometers used by different raters in the same participant. Objective: The aim of this study was to determine the intra-reliability of pressure pain thresholds (PPTs) measured using a digital algometer with and without a digital screen by different raters at the same time in a pain-free population. Methods: Participants were healthy adults. PPTs were assessed using two different algometers: a digital algometer with a digital screen for a feedback of the pressure curve rate (SpTech Digital Algometer); and a digital algometer without a screen (Wagner Instruments FDX-25, Greenwich, CT, USA). Four PPT points were used: upper trapezius, lumbar spine, extensor carpi ulnaris, and tibialis anterior. The Copenhagen Psychosocial Questionnaire II was used to assess burnout, stress, sleeping problems, depressive symptoms, somatic stress, and cognitive stress. The intraclass coefficients (ICCs) for intra-rater reliability was calculated using a two-way mixed effects model, single measurement type, and absolute agreement definition. Results: A total of 47 healthy participants with a mean age of 30.51 (11.35) years were included. The upper trapezius and extensor carpi ulnaris had the lowest PPT values, and the tibialis anterior had the highest PPT value. Females had the lowest PPT values when compared with males with *p* < 0.05 in the upper trapezius and extensor carpi ulnaris regions. The intra-rater reliability ranged from good to excellent reliability, with the ICC values of rater 1 being higher when compared with rater 2. The PPT in tibialis anterior had the highest mean ICC scores. Conclusions: The intra-rater reliability of PPTs measured by different digital algometers ranged from good to excellent reliability. The rater with more experience demonstrated higher reliability.

## 1. Introduction

Chronic pain in Portugal affects one in three adults [1], and the management plan should be designed accordingly with the pain mechanism as nociceptive, neuropathic, and nociplastic [2,3,4,5,6,7,8]. The proposed criteria and grading system to differentiate nociplastic pain from other types of pain involves multiple sorts of hypersensitivities, such as pressure, as a part of this process [9]. The proposed criteria and grading system for distinguishing nociplastic pain from other types of pain include a variety of hypersensitivities, including pressure [9].

Pressure pain threshold (PPT) testing using an algometer is validated, and it is a reliable measurement instrument for pain sensitivity assessment [10,11,12,13]. PPT activates the mechanoreceptors in the deep somatic tissues (muscles, fascia, and joints), which can exhibit sensitivity to mechanical stimuli [14]. PPTs assessed in a localized pain area can reflect localized hyperalgesia, whereas PPTs assessed in areas remote from the painful region reflect widespread hyperalgesia [3,15]. A hyperalgesia of the affected area is thought to reflect peripheral sensitization of Aδ and C fibers, and a widespread lowered PPT may reflect the dysfunction of the endogenous pain inhibition mechanism [14]. To recognize these phenomena in research and in clinical practice, normative cut-off point reference values were calculated, corresponding to the 10th and 25th percentiles from the mean in free-pain populations as the lower PPT limit value for being considered hypersensitive and the 75th and 90th percentiles as the upper PPT limit for being considered hyposensitive [16,17].

In research, the most common algometer used is a digital algometer with different equipment, where the participant gives a verbal cue when they start feeling a pain sensation; others use a hand-held switch for this function, and some have a digital screen for feedback of the pressure curve rate for the practitioner. These are some of the few differences, but the significant difference between different digital algometers is a digital screen to display the pressure curve rate [18].

Moreover, expensive algometers and a lack of normative data between symptomatic and asymptomatic populations have been identified as potential barriers for implementing quantitative sensory testing in clinical practice [19,20]. Regarding PPTs, there are reference values from health participants [16,17] as well as excellent concurrent validity between an analog algometer (less expensive) and a digital algometer (more expensive) by the same rater [18]. Nevertheless, the question remains as to whether different algometers used by different raters at the same time are reliable, taking into consideration different algometers and levels of experience. Also, there is growing evidence that sleep and psychological factors such as stress increase pain sensitization, which should be checked when measuring quantitative sensory measures like pressure pain thresholds [21].

The primary aim of this study was to determine the intra-reliability of pressure pain thresholds measured using a digital algometer with and without a digital screen by different raters at the same time in a Portuguese pain-free population. The secondary aim was to add reference values for the healthy Portuguese population.

## 2. Methods

### 2.1. Participants

This study followed the Quality Appraisal Tool for Studies of Diagnostic Reliability (QAREL) guidelines [22]. Participants were recruited from the Superior Health School Jean Piaget Algarve and Futebol Club Ferreiras, which included students, teachers, staff members, and football players. The inclusion criteria were adults, no pain in the last month prior to the study, and no previous history of musculoskeletal lesions in the neck, lumbar, and limbs in the last year [12,13].

The exclusion criteria were: medical history of cardiovascular or cerebrovascular events; major chronic diseases; neurologic diseases; metabolic diseases; pregnancy; rheumatologic diseases; fibromyalgia; intake of any pain medication for less than 48 h before the procedure; and intake of antidepressants in the last two weeks [16,17].

An external examiner from Superior Health School with more than 15 years of clinical experience conducted a routine clinical examination to determine that the individuals met the aforementioned criteria. This examination included questions about previous history of musculoskeletal lesions, any source of pain, and neck, lumbar, and upper and lower limbs’ range of motion.

This study was conducted in accordance with the Declaration of Helsinki and was approved by the Ethical Council at Institute Piaget (CEIP) with the approval number P11-S20-07/06/2022.

### 2.2. Design

#### 2.2.1. Procedure

Pressure pain thresholds (PPTs) were assessed using two different algometers: a digital algometer without a digital screen for a feedback of the pressure curve rate (Wagner Instruments FDX-25, Greenwich, CT, USA) and a digital algometer with a digital screen (model SpTech, MedOR, Medtech, Santa Catarina, Brazil). The first algometer has already been demonstrated to be a reliable tool in healthy [12] and symptomatic participants [12,23], while the second algometer has been employed in research [24].

Both devices were calibrated according to the manufacturer’s instructions before the measurements. They consisted of a 1 cm^2^ rubber tip applicator, placed perpendicularly to the skin and mounted on a force transducer at an application rate of 1.0 kg/s. The participants were instructed to say “yes” as soon as the pressure sensation changed to pain sensation; at that time, the rater immediately removed the algometer and recorded the PPT value. PPT was defined as the minimum pressure required to elicit a pain response. A maximum cut-off pressure of 1000 kPa was used. PPTs were measured three times with a 10 s interval at each point, and the average value was used for statistical analysis. There was a 30 s interval between each PPT point measurement to minimize wind-up [25].

Four PPT points were used: upper trapezius, lumbar spine, extensor carpi ulnaris, and tibialis anterior. The upper trapezius point was localized in the midpoint between C7 and acromion, [26,27,28]; the lumbar spine was tested at the erector spinae, 2 cm lateral to the L4/L5 interspinous space [16]; the extensor carpi ulnaris muscle belly point was localized from the lateral epicondyle that was the reference point, 40 mm inferior in a vertical line and then 20 mm posterior [26,27,28,29]; and the tibialis anterior point was defined approximately 2.5 cm lateral and 5 cm inferior to the tibial tubercle [12,27,28]. The participants were in prone position for the upper trapezius and lumbar spine and then changed to supine position for extensor carpi ulnaris and tibialis anterior measurements. Each PPT localization was marked by a pen marker; for normalization, the assessment was conducted on the dominant side of the participant. The main reasons for the chosen muscle PPT points were based on the existence of normative cut-off points reference values [16,17] and typical PPT points on the neck, lumbar, and upper and lower limb area [26,27,28,29].

Rater 1 always used the algometer from Wagner Instruments FDX-25, and rater 2 used the SpTech Digital Algometer. The difference from both algometers was a digital screen to display the pressure curve rate [18]. Rater 1 was an osteopath with 19 years of clinical experience, 6 years of experience using an algometer, extensive training in PPTs, and previous publications using an algometer [27,28]; and rater 2 was an osteopath and physiotherapist with 15 years of clinical experience and 2 years of experience using an algometer only in clinical practice, without any experience in previous research. Repeat measurements were used to assess intra-rater reliability by one rater on two different days (R1D1 vs. R1D2). Rater 1 commonly used their algometer in research and in clinical practice, and rater 2 only used it in clinical practice and was not used to measuring PPT without feedback from the pressure curve rate. These were the main reasons why algometers were not changed between raters, which could affect the intra-reliability.

Both raters examined participants twice throughout the first session. Furthermore, demographic information was acquired, and individuals filled out the Copenhagen Psychosocial Questionnaire II. The second session, held a week later, solely comprised PPT measures. To ensure blindness among raters and consistency with past findings, each rater recorded the measurements on an empty sheet on days 1 and 2. A senior researcher, who was unaware of the rater allocation, examined the data.

#### 2.2.2. Experimental Protocol

All PPT points were marked by rater 1, who has experience in previous studies [27,28]. PPTs were measured twice by both raters in each session. The sequence of the procedure was as follows: PPT was measured in the upper trapezius, in the lumbar spine, in the extensor carpi ulnaris, and lastly in the tibialis anterior. After rater 1’s assessment, participants were allowed to rest comfortably for 15 min prior to rater 2 entering the room and repeating the same protocol. The protocol was repeated at the same time, separated by one week.

### 2.3. Demographic Data

The following variables about the participants were collected: gender, age, height, weight, and body mass index (BMI).

### 2.4. Psychological and Health-Related Variables

The long version of the Copenhagen Psychosocial Questionnaire II (COPSOQ II) is designed to assess psychosocial factors, health, and wellbeing, and it is composed by 128 items of standardized self-reported belonging to 41 scales that represent seven domains [30]; the Portuguese version was validated by [31]. In this study, the domain of health and wellbeing was used, which is composed of six scales: burnout (4 questions), stress (4 questions), sleeping problems (4 questions), depressive symptoms (4 questions), somatic stress (5 questions), and, cognitive stress (4 questions); general health was assessed by 1 question, which means there were 26 questions in total. Questions were scored on a five-point Likert scale ranging from 1 (not at all) to 5 (all the time), except for the general health question that ranged from 1 (excellent) to 5 (poor). The scale scores were calculated as an average of the items included. Each scale has a good internal consistency between 0.7 and 0.9 in the Portuguese population [32]. For easier reading and interpretation, sleeping troubles will be mentioned as sleep. This questionnaire was previously used in research with quantitative sensory testing in healthy participants [27].

### 2.5. Statistical Analysis

G*Power 3.1.9.2 software for Mac OSX [33] was used to calculate the required sample size with a power of 80% and a two-sided t-test, with =0.05 giving 45 participants.

Descriptive statistics were calculated for gender, age, height, weight, body mass index (BMI), and for the psychological and health-related variables (COPSOQ), they were presented as the mean and standard deviation (SD) for continuous variables and as the percentage for categorical variables. The Shapiro–Wilk test was used to test the normal distribution of the variables.

The intra-rater reliability for algometry was examined using intraclass correlation coefficients (ICCs). ICCs were calculated using a two-way mixed effects model, single measurement type, and absolute agreement definition [34,35]. ICC estimates and their 95% confidence intervals were reported. ICCs were interpreted as follows: less than 0.50—poor reliability; between 0.50 and 0.75—moderate reliability; between 0.75 and 0.90—good reliability, and greater than 0.90—excellent reliability [34].

Mean differences between PPT measurements were analyzed with an independent samples t-test. Pearson’s product moment correlation was used to assess the associations between PPT with COPSOQ. All tests were conducted bilaterally, with a statistical significance threshold of 0.05. The acquired data were statistically analyzed using IBM^®^SPSS^®^ version 25 software.

## 3. Results

**Figure 1:**
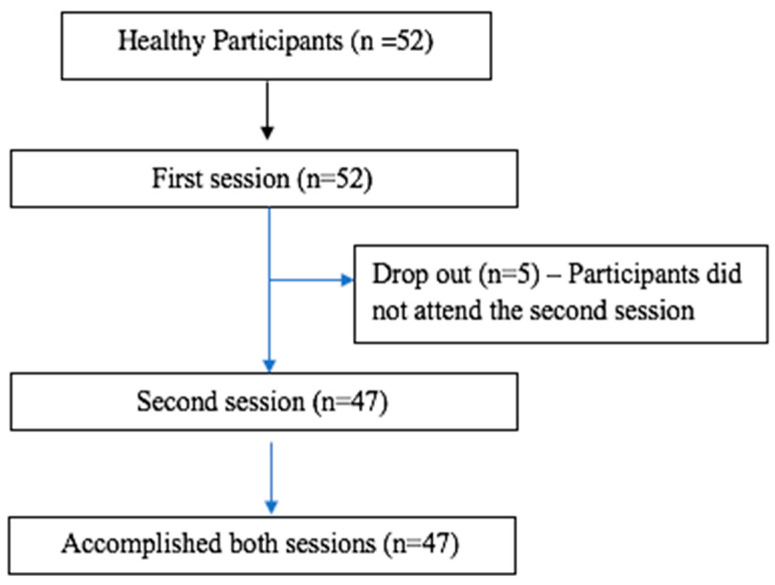
Flowchart diagram of the office.

This study included 52 healthy participants that fulfilled the inclusion criteria and completed the first session. Five participants dropped out prior to the second session, and so, 47 participants accomplished both sessions and were included in the final statistical analysis (Figure 1).

The participants’ characteristics are summarized in Table 1. There was almost a perfect distribution for gender, and the mean age was 30.51 (11.35). In relation to the COPSOQ questionnaire, stress was the health-related variable with the highest scores and depression symptoms was the health-related variable with the lowest scores.

In Table 2, the mean PPT values from the four measurements are presented (rater 1 plus rater 2). The upper trapezius and extensor carpi ulnaris had the lowest PPT values, followed by lumbar spine and tibialis anterior, which had the highest PPT value. Females had the lowest PPT values when compared with males with a statistical significance in the upper trapezius and extensor carpi ulnaris.

The intra-rater reliability of algometry is shown in Table 3, ranging from good to excellent reliability. The ICC values of rater 1 were higher when compared with rater 2, achieving an excellent intra-rater reliability in all PPT points. The PPT in tibialis anterior had the highest mean ICC scores. The mean PPT difference was lower in the extensor carpi ulnaris and highest in the tibialis anterior.

Table 4 demonstrates the correlation between COPSOQ health variables with PPTs. The variable stress had a negative significant correlation with upper trapezius r(45) = −0.297, *p* < 0.05 and extensor carpi ulnaris r(45) = −0.327, *p* < 0.05 PPTs.

## 4. Discussion

The aim of this study was to determine the intra-reliability of PTT measured using a digital algometer with and without a digital screen by different raters at the same time in a pain-free population. This study demonstrated that the intra-rater reliability of PPT ranged from good to excellent reliability.

The reliability results of the current study are in accordance with previous and recent studies from Castien et al. [18], Tabatabaiee et al. [36], and Jayaseelan et al. [37] and also with a recent systematic review [38]. Also, from Castien et al. [18], there was no difference in the reliability between an analog with a digital algometer from a single rater, but the current study adds reliability for different digital algometers with different raters at the same time. This is important for clinical practice regarding the variety of different algometers available to measure PPT in order to compare the normative cut-off point to verify a hyposensitive or hypersensitive PPT [16,17]. Mainly, the higher ICC values found for the tibialis anterior muscle are important as a remote point for assessing widespread mechanical hyperalgesia [3,15] in most musculoskeletal conditions, such as chronic low back pain and neck pain [39,40,41]. Recent systematic reviews found widespread pressure hyperalgesia at the tibialis anterior in chronic neck pain [38,39] and in chronic low back pain [41].

Moreover, the algorithm proposed to recognize nociplastic pain in clinical practice, which included sensitivity to pressure [9]. The definition of nociplastic pain implies an inference of central sensitization and does not exclude a peripheral sensitization [40]. Central sensitization can be assessed through quantitative sensory tests, with the principal hallmarks in clinical practice being allodynia, secondary hyperalgesia, and widespread mechanical hyperalgesia [14,15]. PPT measurements by algometry in local and remote points have been proven to be a reliable method [18,36,37,38], even with different algometers on the same participant, as this study demonstrated. Nevertheless, there were mean differences in PPT values between both raters, and when analyzing the standard deviations from the mean values and comparing with PPT normative values, there were health participants with hypersensitive PPT values. Therefore, this variability in the clinical setting needs to be integrated with clinical history, signs, and symptoms [9].

The intra-rater reliability was higher in all PPTs for rater 1, who has more experience in using algometry in clinical practice and research. This finding is consistent from Królikowska et al. [35], in which experience is a crucial factor in improving reliability. However, it seems the algometry reliability in musculoskeletal pain is also associated with clinical experience. The study from Stausholm et al. [42] included three raters, two with 5 and 18 years of clinical experience and only 30 min of algometry training and the third rater with 1 year of clinical experience but ongoing practice in algometry in a previous trial. The rater with more clinical experience achieved the highest score in intra-rater reliability, despite all three raters achieving excellent intra-reliability. Another study with final physiotherapist students receiving eight hours of algometry training demonstrated good to excellent intra-reliability [43]. Interestingly, there were no intra-reliability differences in this study from the two digital algometers used by the same rater, an equal Wagner algometer and a Somedic digital algometer with a digital screen for feedback of the pressure curve rate and with a hand-held switch for the participant to press when they feel the first pain sensation [43]. Therefore, clinical experience and training in using an algometer are perhaps the most crucial important aspects and can clearly compensate for the absence of an algometer without a digital screen to display the pressure curve rate [18].

The second aim of this study was to add reference values for the Portuguese healthy population, despite the limited sample size for this study type. Nonetheless, when compared with other studies with a larger healthy non-Portuguese population, the mean PPT values for the upper trapezius [16,17], lumbar spine, and tibialis anterior [17] were similar, particularly for the female gender. In studies conducted in healthy Portuguese office workers, the PPT values were almost equal for the upper trapezius, extensor carpi ulnaris, and tibialis anterior [28]. As a result, the normative cut-off point values from the aforementioned research may be utilized to quantify hypersensitivity to mechanical pressure, which is an important finding for distinguishing between a local and distal hyperalgesia [3,15].

Furthermore, the mean PPT values were lower in females compared with men in all PPTs, which is in accordance with the literature for the healthy population [16,17,18,44]. Recently, for the first time, reference values of the abdominal area were established, and female participants in all group ages demonstrated an enhanced sensitivity to PPT in the lower and upper quadrants of the abdominal area [45]. This is also present in different musculoskeletal conditions where the PPT is lower in females when compared with males in knee osteoarthritis [46], tension-type headache, and migraine [47] and in adolescents with chronic pain [48]. The lower values in PPTs can be explained by several studies investigating conditioned pain modulation demonstrating a lower pain inhibition in healthy female subjects when compared with male healthy subjects [49,50,51,52].

Finally, despite it being small, there was a significant negative correlation between stress and upper trapezius and extensor carpi ulnaris PPTs. There is growing evidence that stress causes pain sensitization [53,54], and from a systematic review, stress was a longitudinal psychological factor associated with pain intensity in persistent musculoskeletal pain [21]. The study from Hven et al. [54] used PPTs to measure perceived stress. Their findings were equal to our study in the trapezius muscle, with an association of higher levels of stress with lower values of PPTs in men and women. Nevertheless, they concluded that there was poor discrimination from the algometry in differentiating individuals with different stress levels [54]. In our opinion, the usefulness of an algometer is not to recognize the stress level; there are plenty validated questionnaires to assess stress, such as the COPSOQ II used in the present study, but they help to recognize the pain profile of a chronic presentation in the clinical setting [9].

### 4.1. Clinical Implications

Different algometers are reliable for determining pressure pain thresholds in the healthy population. The algometers utilized in the current study were both with and without a digital screen providing feedback on the pressure curve rate, and they were regarded as low-cost algometers when compared with more sophisticated models. In the recent decade, research has provided reference PPT values from the healthy population [16,17], a vital step for health clinicians in determining if a PPT is hypersensitive or a signal of local or central sensitization based on its localization with respect to the painful location [3,15].

### 4.2. Study Limitation

In the current study, it was not possible to measure PPTs on the same workweek day for all the participants. The burnout scale was the second with the highest scores, and fatigue resulting from daily activities can influence the QST results, so it might be considered a limitation [55].

The phase of the menstrual cycle is a confounding factor for pain sensitivity that was not controlled for in the present study [56]. Nevertheless, the sample size was equally distributed between genders, and this has a higher effect when the sample size is mainly composed of female participants.

The current study did not use randomization for both devices and raters to assess inter-rater reliability [35]. As a result, future research should look into inter-rater reliability utilizing different types of algometers.

### 4.3. Conclusions

The intra-rater reliability of pressure pain threshold measured by different digital algometers ranged from good to excellent reliability. The rater with more experience demonstrated excellent intra-rater reliability and used an algometer without a digital screen for pressure curve rate feedback. The tibialis anterior point was the PPT point with higher intra-reliability, with both raters achieving excellent intra-reliability. Female participants had lower pressure pain threshold values with a statistical difference in upper trapezius and extensor carpi ulnaris. The variable stress had a negative significant correlation with pressure pain threshold in the same mentioned points.

## Figures and Tables

**Table 1:** Participants’ characteristics.

Characteristic	Mean (SD)	N	%
Age (years)	30.51 (11.35)		
Gender			
Female		24	51.1
Male		23	48.9
Weight (kg)	70.29 (15.25)		
Height (cm)	167.49 (10.58)		
BMI (kg/m^2^)	24.90 (4.20)		
**Copenhagen Psychosocial Questionnaire II**			
Sleep (1–5)	2.13 (0.90)		
Burnout (1–5)	2.33 (0.74)		
Stress (1–5)	2.55 (0.67)		
Depression symptoms (1–5)	1.78 (0.60)		
Somatic stress (1–5)	2.02 (0.69)		
Cognitive stress (1–5)	2.19 (0.76)		

**Table 2:** Mean pressure pain point values (kPA).

Pressure Pain Points	All Sample (n = 47)	Female (n = 24)	Male (n = 23)	*p* Value
	M ± SD	M ± SD	M ± SD	
Upper Trapezius	240.42 ± 99.93	210.36 ± 88.83	271.79 ± 102.03	0.34 *
Lumbar Spine	416.60 ± 172.51	374.54 ± 125.21	460.48 ± 204.68	0.88
Extensor carpi ulnaris	237.67 ± 93.53	209.47 ± 85.45	267.09 ± 94.26	0.33 *
Tibialis Anterior	440.25 ± 176.14	394.12 ± 150.26	488.38 ± 191.11	0.66

* *p* < 0.05.

**Table 3:** Intra-rater reliability of the algometry assessment.

Pressure Pain Points	Rater	ICC	95% CI	Mean Difference in kPa (SD)
Upper Trapezius	1	0.93	0.88, 0.96	
	2	0.87	0.77, 0.93	37.34 (50.7)
Lumbar Spine	1	0.94	0.89, 0.96	
	2	0.85	0.73, 0.91	43.02 (79.9)
Extensor carpi ulnaris	1	0.92	0.86, 0.95	
	2	0.84	0.71, 0.91	9.20 (54.1)
Tibialis Anterior	1	0.93	0.88, 0.96	
	2	0.90	0.83, 0.94	45.74 (75.9)

Legend—CI, confidence interval; ICC, intraclass correlation coefficient; SD, standard deviation.

**Table 4:** Pearson correlation COPSOQ with pressure pain threshold points.

Variables	Pressure Pain Points			
	Upper Trapezius	Lumbar Spine	Extensor Carpi Ulnaris	Tibialis Anterior
Sleep (1–5)	−0.039	−0.048	−0.142	0.058
Burnout (1–5)	−0.194	−0.228	−0.287	−0.076
Stress (1–5)	−0.297 *	−0.253	−0.327 *	−1.51
Depression symptoms (1–5)	−0.231	−0.160	−0.272	−0.133
Somatic stress (1–5)	−0.182	−0.163	−0.229	−0.024
Cognitive stress (1–5)	0.025	0.021	−0.051	0.135

* *p* < 0.05.

## Data Availability

The data are available upon request from the corresponding author.
